# Improving Willingness to Care and Training Needs for PLWHA from the Perspective of Student Nurses in China: A Qualitative Study

**DOI:** 10.3390/healthcare12161646

**Published:** 2024-08-18

**Authors:** Chunhong Shi, Jerome V. Cleofas

**Affiliations:** 1 Nursing Department, XiangNan University, Chenzhou 423000, China; shichunhong1987@163.com; 2 College of Nursing and Allied Health Sciences, St. Paul University Manila, Manila 1004, Philippines; 3 Department of Sociology and Behavioral Sciences, De La Salle University, Manila 1004, Philippines

**Keywords:** HIV, AIDS, nursing education, PLWHA, student nurses, training needs, willingness to care

## Abstract

People living with HIV and AIDS (PLWHA) deserve equitable and high-quality care. Current HIV and AIDS nursing education may not adequately prepare student nurses for the complexities of caring for PLWHA, and the perspectives of student nurses have not been sufficiently revealed in nursing education research. This study aimed to explore the viewpoints of student nurses with AIDS care experience on methods to improve their care willingness for PLWHA and to identify their educational and training needs. A descriptive qualitative study design was employed, interviewing 18 undergraduate student nurses from 14 tertiary hospitals across 7 provinces in China. Content analysis of transcripts revealed insightful suggestions for improving nursing students’ willingness, such as increased HIV and AIDS education and training, psychological preparation, positive role modeling, raising awareness about AIDS patients, and fostering nursing professionalism. Highlighted education and training needs include progress in HIV and AIDS treatment, preventive measures, psychological support for PLWHA, post-exposure protocols, and HIV-infected risk behaviors. These findings highlight the need for HIV and AIDS education, psychological support training, and stigma-reduction strategies. This study provides valuable insights that could inform policymakers, educators, and healthcare providers on preparing future nurses to meet the complex needs of PLWHA.

## 1. Introduction

Now in its fourth decade, the human immunodeficiency virus (HIV) epidemic requires great global efforts to reverse and control its spread, increase healthcare access, and strengthen the role of nurses in caring for people living with HIV and AIDS (PLWHA). However, PLWHA continue to endure the stigma and discrimination coming from the general public and even from healthcare providers [1,2], which make it difficult for them to access both general healthcare and mental health services [3]. Stereotypes surrounding PLWHA adversely affect patients’ treatment outcomes [4] and well-being [5]. To overcome these stereotypes and negative influences, it is imperative to implement stigma-reduction interventions, advocate for policies that protect the rights of PLWHA, and foster a culture of inclusion and respect. Furthermore, more needs to be done to ensure that healthcare providers are equipped with the knowledge and skills to provide compassionate and non-judgmental care [6]. Nurses, who make up a large proportion of healthcare providers, are critical to the successful treatment and control of HIV infection [7,8]. However, multiple studies demonstrate that many registered nurses are reluctant and unprepared to provide care to patients with HIV due to limited knowledge, misconceptions, and fear of infection [9,10], as well as stressful work situations [11]. Nursing students become registered nurses after completing personal, professional, and organizational transitions [12], and they will most likely be allocated to care for PLWHA throughout clinical practice. Thus, their willingness to care for PLWHA greatly impacts the quality of care they provide.

In optimizing HIV-related clinical outcomes, educational policies and interventions are needed for nursing students to improve their willingness to provide quality HIV treatment and care [13,14]. Indeed, nursing students who have accurate HIV/AIDS knowledge and maintain positive attitudes towards PLWHA are more likely to exhibit a stronger willingness to care, as well as to protect themselves from contracting HIV [15,16]. Classroom and clinical teaching regarding HIV- and AIDS-related concepts [17], prevention and stigma reduction [18] is extremely important for improving the care willingness of nursing students. Previous studies have also shown that fostering high professional commitment and empathy among nursing students can improve their willingness [19]. Additionally, it is important to improve nursing students’ perceptions of HIV-infected patients and increase their clinical practical experiences in healthcare settings [20]. Similarly, nursing students’ perceived educational needs regarding sex workers are positively correlated with their willingness to provide care for this population [21]. In Africa, health professional training institutions have crafted an array of interdisciplinary, collaborative, and case-based HIV training programs to bolster the proficiency in HIV care among newly graduated healthcare providers [22]. Studies have claimed that narrative photography [23], patient testimonials, role playing, gaming, information sharing, and peer education strategies are promising interventions for reducing HIV-related stigma [24,25]. Furthermore, integrating courses on nurse-initiated antiretroviral therapy management into undergraduate nursing programs appears to be beneficial for new nurses in managing PLWHA [26,27].

Nursing faculty are responsible for preparing every nursing student to professional nurses with HIV competencies. Although educational policies and clinical training play a significant role in imparting knowledge about HIV and AIDS, the effectiveness of these programs in improving the care willingness of nursing students remains a concern. Previous studies have highlighted large knowledge gaps among nursing students in the transmission and management of HIV [28,29] and their unpreparedness in their role to care for PLWHA [30]. Further exploration is required to identify the specific training needs and educational interventions for improving their willingness. Nursing students’ perspectives on such an issue could offer valuable insights into the methods to improve their willingness and the training they require to feel competent in HIV-related care. Most research has focused on the experiences of nursing students in providing care to PLWHA [22,30], as well as their feelings and attitudes towards PLWHA [20]. However, few studies have explored their suggestions for improving the willingness of nursing students to care for PLWHA and their HIV and AIDS training needs. This study sought to address this gap by drawing qualitative insights from interviews with student nurses with AIDS caregiving experience. Specifically, the study objectives were to (1) examine students’ perspectives on improving willingness to care for PLWHA and (2) describe their perceived training needs related to HIV and AIDS care.

## 2. Materials and Methods

### 2.1. Study Design

Descriptive qualitative design was used in this study. In nursing research, descriptive qualitative research design is a common method used to describe and understand the experiences, behaviors, and perspectives of individuals or groups [31]. This design is particularly suitable for exploring new topics or gaining insights into complex issues. We conducted in-depth interviews with student nurses to gather rich qualitative data, which were then analyzed using content analysis to identify themes related to the care willingness and training needs of student nurses in HIV and AIDS care.

### 2.2. Participant Selection

The following were the inclusion criteria for student nurses in this study: older than 18 years old; full-time undergraduates; cared for one or more patients with AIDS; and provided informed consent to participate in this study. Part-time students, junior college students, or graduate students without experience in caring for patients with AIDS were excluded. Recruitment was concluded upon reaching the point of data saturation, wherein the collected data can no longer produce new themes [32]. This study followed the reporting criteria for qualitative research [33].

### 2.3. Data Collection

On the Yiqixiu platform (a multifunctional tool with a large number of templates facilitating the design of invitations), we have designed an advertisement aimed at recruiting potential participants. The advertisement clarified the inclusion and exclusion criteria, interview duration, process, potential risks, benefits (compensated with RMB 100, equal to approximately USD 13.78), and research objectives. We shared the advertisement link via QQ and WeChat social platforms and invited teachers from institutions such as Xizang University and Guangxi University of Traditional Chinese Medicine, as well as student nurses, to help spread the recruitment advertisement and expand our reach. Potential participants were invited to fill out an online form or contact the research team by phone or email. To ensure a diverse sample, we screened respondents based on variables such as gender, age, university attended, and internship hospital.

From 16 July to 25 August 2021, semi-structured, in-depth telephone interviews were conducted with student nurses utilizing purposive sampling. Before the interview, participants were asked to provide general information and signed an informed consent form. Phone interviews were performed in a quiet and comfortable place (study room or bedroom) to avoid interruptions. The recording equipment (a tape recorder and a mobile phone) was used to record the interviews. Based on the existing literature [34,35] and the research team’s discussions, we formulated the initial interview questions, specifically inquiring about improving student nurses’ willingness to care for PLWHA and their desired HIV and AIDS training content. Following a pilot test with two student nurses, we engaged in an iterative process to refine the questions, incorporate feedback, and structure them into a more comprehensive and coherent framework. As a result, we created a formal interview outline (Table 1) to gather the essential data for our research objectives. Important words and expressions of the participants (e.g., laughter, quiet, etc.) were recorded during the interview. Each interview lasted approximately 30 min.

### 2.4. Ethics Consideration

This study was approved by the Medical Ethics Committee of Xiangnan University (No. KY-201808010) on 1 December 2018 and by the Ethics Committee of St. Paul University, Manila (No. 2021-045-IGS-CNA), on 24 June 2021. The rights and willingness of student nurses were fully considered, and they could withdraw from the research at any time without any reason. Participants were informed about their potential interests as well as the research process and purpose. Researchers strictly abided by the provisions of the 1995 Helsinki Declaration (revised by Edinburgh in 2000), including the principles of autonomy, confidentiality and non-harm. Upon completion of the interview, all participants were provided a stipend of approximately USD 13.78.

### 2.5. Data Analysis

The interview data were transcribed verbatim into Chinese transcripts within 48 h by the interviewer (the first author) to minimize potential memory decay effects. The transcripts also included detailed descriptions of the respondent’s interaction process and non-verbal cues, such as pauses, laughter, and gestures, which provide important contextual information for the analysis. Each transcript was double-checked by a second interviewer who was not involved in the original interview process. NVivo software version 11(QRS International) (https://qsr-nvivo.software.informer.com/11.0/ accessed on 1 June 2024) was used to assist with qualitative data analysis. Qualitative content analysis was used to analyze the data [36]. The specific steps were as follows: (1) decontextualization: read the transcript of the interview repeatedly and carefully until the whole meaning is obtained; (2) recontextualization: break down the information, analyze it line by line, identify meaningful statements, and code them; (3) categorization: code and categorize recurring statements to established categories; (4) compilation: find connections between categories to generate themes; (5) and continue until saturation is reached, i.e., no new themes or subordinate themes occur.

## 3. Rigor

To ensure the rigor of the study, the following four criteria [37] were adopted: (1) credibility: all transcripts were double-checked, and data analysis was carried out independently by two researchers. The consistency of the results was examined discussed by the research team. (2) Dependability: to avoid the loss of textual information, the researchers used memos to track coding process and audit trails were documented. (3) Transferability: student nurses from 14 tertiary hospitals across seven provinces were used to maximize the heterogeneity of the sample and increase the transferability. (4) Confirmability: the results were returned to the participants for validation.

## 4. Results

### 4.1. Demographic Characteristics

This study finally interviewed 18 student nurses from 9 schools of nursing, with internships at 14 hospitals across 7 provinces. The majority of the participants were female (n = 14, 77.78%), aged between 21 and 23 years. They cared for between one to eight patients with AIDS (median: 2, interquartile range: 0.5) in a range of clinical departments, such as outpatient services, oncology, gynecology, emergency care, neurosurgery, thoracic surgery, intensive care units, cardiovascular medicine, cardiothoracic surgery, neurology, obstetrics, gastrointestinal surgery, and other specialized units. The student nurses had received AIDS-related training between zero and three times (median: 1, interquartile range: 1). Table 2 shows demographic information on the participants.

### 4.2. Methods to Improve Care Willingness and Training Needs of HIV and AIDS Education

The transcripts were analyzed and categorized into four subordinate themes and fourteen subcategories. Table 3 shows a summary of the results.

### 4.3. Student Nurses’ Willingness to Care for PLWHA

*Expressions of care willingness*

We explored student nurses’ willingness to care for PLWHA before and after internship, focusing on two subcategories: “unexpecting to care for PLWHA before internship” and “strong willingness to care for PLWHA in future work”.

Unexpecting to care for PLWHA before internship

Ten participants stated that they did not expect to encounter HIV patients and were unprepared to care for HIV patients prior to their clinical internship.

“*I didn’t even consider [taking care of AIDS patients during my internship] in school. I was surprised when I first came across AIDS patients in clinical practice.*”

(P15)

“*I had never considered caring for people with AIDS, and students who choose this [nursing] major in school rarely anticipate actually caring for patients with this [AIDS].*”

(P5)

Strong willingness to care for PLWHA in future work

All participants expressed that they would make efforts to provide fair and quality care for PLWHA in their future work, as it is a nurse’s unassailable professional responsibility to deliver services to all patients.

“*As a clinical nurse, I would be willing to provide (care) because AIDS patients are also patients, and providing care is part of our job.*”

(P5)

“*I believe that because I am a nurse, I am open to treating and caring PLWHA in my future clinical work.*”

(P18)

*Proposed interventions of improving care willingness*

Participants shared a variety of valuable suggestions based on their personal experiences to increase nursing students’ willingness. This subordinate theme had five major subcategories: “increase HIV and AIDS education and training”, “introduce psychological preparations”, “set positive examples”, “raise awareness of AIDS patients”, and “foster nursing professionalism”.

Increase HIV and AIDS education and training

Thirteen participants suggested that HIV and AIDS education and training could improve students’ related knowledge, reduce HIV-related discrimination and stigma, and increase nursing students’ willingness to provide services to PLWHA.

“*To improve theoretical knowledge and practical training by simulating scenarios, for instance, what should be done in the clinical care of an AIDS patient.*”

(P8)

“*Working on educating nursing students… I believe standardized operations can reduce the occurrence of needle-stick injuries and occupational exposure.*”

(P16)

Make psychological preparations

Ten participants agreed that schools and hospitals should encourage students to prepare care for AIDS patients from the heart.

“*Psychologically, only nursing students believe that AIDS is not so frightened as long as they practice appropriate self-protection, they will say “I do want to care for AIDS patients.*”

(P16)

“*I believe that encouraging students to develop proper psychological awareness of caring for AIDS patients in school and clinical settings can help them care for PLWHA.*”

(P18)

Set positive role models

Students (n = 7) stated that positive reporting and experience sharing, reducing incorrect perception and stigma associated with AIDS patients, may encourage students to volunteer caring for PLWHA.

“*Nursing students’ willingness can also be increased by showing videos of health care workers who have successfully helped AIDS patients gain hope for their lives.*”

(P2)

“*Seniors may share their clinical experiences, inform students of what they learned in caring for AIDS patients and share that AIDS patients are not as frightening as they believe.*”

(P1)

Raise awareness of PLWHA

Six participants stated that promoting students to learn more about real AIDS cases in clinical settings through scenarios, personal contact with AIDS patients, and experience sharing would increase students’ willingness.

“*Let nursing students know that AIDS patients are not as frightening as they image, just like the aunt I met during my internship, who was upbeat and cheerful.*”

(P4)

“*I believe make students video-link with PLWHA, or read autobiographies written by AIDS patients… Allowing students to immerse themselves in the lives of PLWHA and truly get to know them.*”

(P15)

Foster nursing professionalism

Four respondents believed that schools should emphasize students’ professionalism to encourage them to transcend any challenges, such as delivering care to AIDS patients.

“*Those who work in hospitals must have a sense of mission and responsibility… Caring for PLWHA is the responsibility of every medical professional worker…*”

(P17)

“*…Inspire compassion in student nurses and prepare them with the responsibility to care for AIDS patients.*”

(P10)

### 4.4. HIV and AIDS-Related Educational and Training Programs

*Inadequate HIV and AIDS education and training*

Student nurses believe that AIDS education and training are provided infrequently in both schools and hospitals, with a lack of hands-on AIDS training in schools and specialized AIDS training in hospitals.

Lack of hands-on HIV and AIDS training in school

Schools provide some HIV and AIDS knowledge in the infectious disease curriculum, but not enough nursing skills.

“*In school, we learned some knowledge about AIDS when we studied infectious diseases. In addition, the Red Ribbon Society distributed pamphlets on AIDS prevention and control.*”

(P17)

“*I attended HIV- and AIDS-related lectures once in school, in which they talked about HIV infection and how to avoid it. It was not helpful… there was no practical training…*”

(P8)

Lack of specialized HIV and AIDS training in hospital

Most students noted that the hospital where they interned did not provide a special AIDS lecture or training, but universal precautions were included in occupational exposure, infection control, or pre-service training. Three students reported receiving no HIV/AIDS training throughout their clinical internship.

“*As an intern, I attended an infection control conference where the speaker mentioned AIDS, although he only covered some of it.*”

(P10)

“*The hospital didn’t offer HIV/AIDS lectures. Before starting work, we underwent a week of pre-service instruction on routine safety precautions.*”

(P17)

*Salient HIV and AIDS education and training needs*

Students expressed salient HIV and AIDS education and training needs. Each subcategory represents an aspect of HIV and AIDS knowledge that students want to learn.

Progress in HIV and AIDS treatment

Antiretroviral medications and HIV treatment can slow the HIV and AIDS epidemic. Nearly half of students (n = 8) are interested in AIDS treatment, especially antiretroviral therapy (ART), cost, tolerability, and newer drugs.

“*Has there been a medicinal breakthrough for AIDS treatment in recent years?*”

(P16)

“*I’m more interested in… [I] seem to have heard that a new drug…and I want to learn the treatment dynamics of this disease.*”

(P3)

HIV-prevention strategies

Seven participants stated that lectures or seminars should include emphasize personal protection measures including condom use, hand washing, and PPE.

“*Nowadays, people may be more open about sex, and knowing how to protect oneself is critical [for HIV prevention]… [I] am still interested in specific prevention measures.*”

(P13)

“*[I also want to know] how to prevent AIDS; it would be fantastic if we could treat AIDS with dietary therapy or gymnastics… But it appears that we can’t yet].*”

(P6)

Psychological support skills for PLWHA

PLWHA are prone to psychological discomfort and cognitive impairment, requiring psychological support. Seven respondents wanted to learn more about PLWHA psychological support criteria or recommended practices.

“*I’d like to know how to provide psychological therapy to HIV-positive patients, earn their trust, and make them willing to face the disease positively.*”

(P17)

“*Because AIDS is incurable, the [lecture or seminar] can also include psychological treatment for AIDS patients.*”

(P6)

HIV post-exposure reporting protocols and prophylaxis

Blood exposure accidents require proper response to decrease risk. Six participants did not know how to report HIV-transmission-risk accidents.

“*I’ve learnt preventative measures for occupational exposure, but I’m unclear about monitoring, reporting, and handling infectious disease exposure.*”

(P10)

“*Our lecturers didn’t tell us what to do if we received a needle-stick injury—an occupational exposure—while caring for an AIDS patient… We may need to understand the reporting chain and post-exposure prophylaxis.*”

(P8)

HIV-infection risk behaviors

Risk exposures or behaviors for HIV include sharing syringes for drug use or unprotected sex behaviors. Three participants wanted to know the high-risk behaviors for contracting HIV.

“*I’d also like to learn more about the characteristics of the citizens at high risk of HIV.*”

(P5)

“*I’m most curious about the high-risk behaviors for contracting HIV.*”

(P2)

## 5. Discussion

This qualitative study uniquely examines Chinese student nurses’ willingness to care for PLWHA, revealing strategies to enhance it as well as training gaps. Initially unexpected, their shift to a strong willingness to care post-internship emphasizes HIV and AIDS clinical practice, psychological preparation, positive role modeling, and nursing professionalism. Furthermore, our findings highlight the need for hands-on training and specialized instruction in HIV and AIDS, including updates on treatment, prevention, psychological support, and risk reduction strategies. These insights are crucial for informing policymakers, educators, and healthcare providers in preparing nursing students for HIV and AIDS care.

More than half of participants claimed that they had not expected to encounter PLWHA prior to the internship. This could be attributed to PLWHA’s high level of concealment of their HIV status from others [38,39], inadequate HIV-related knowledge [40] and a lack of educational resources related to HIV and AIDS [41]. Otherwise, we found that there were positive changes after caring for HIV patients. In the transcripts, many students had misconceptions and stereotypes about AIDS patients before delivering care to them, believing that AIDS patients had distinct characteristics such as being emaciated, seriously ill, and so on. However, the care-giving experience informed them that most AIDS patients do not have such characteristics, and that many are cheerful, loving, and just like other patients. This is consistent with the findings of Kurniawan et al. [42] in Indonesia, which showed that nurses shifted from negative perceptions to a positive attitude and developed a greater understanding of their patients following direct interaction. Chan et al. [43] also found that primary health care providers who have cared for an AIDS patient or had HIV-positive relatives were more likely to care for and help PLWHA. It is recommended that nursing education programs integrate more practical experiences or simulated and real-world interactive modules with PLWHA to develop favorable attitudes towards HIV and AIDS care among student nurses.

Participants indicated that increasing HIV and AIDS education and training was an effective strategy for increasing students’ willingness to provide services, which was consistent with the findings of Machowska et al. [18] in India. Educational programs or lectures on HIV and AIDS can fill knowledge gaps [44] and reduce stereotypes or prejudices about PLWHA [45]. Chan et al. [43] reported that less discrimination against PLWHA was observed among primary healthcare providers who received more training in HIV and AIDS prevention and treatment. More HIV and AIDS knowledge is associated with a more positive attitude toward PLWHA [46,47]. Healthcare providers’ attitudes and confidence in delivering care to PLWHA also increased with sufficient education [48]. Unfortunately, scholars have discovered a current lack of HIV education programs in nursing education [49] and an urgent need to improve HIV and AIDS theoretical knowledge and practical skills for educators and students. Moreover, the faculty would employ various methods (e.g., narrative photography, peer education) to practically and mentally prepare students to work with PLWHA [50,51]. “Setting positive role models” is another technique to boost student nurses’ willingness to care for PLWHA. Previous studies have highlighted the importance of role models in nursing education, emphasizing the need for nurse educators and clinicians to foster a culture of professional nursing care [52,53,54]. Unfortunately, nursing faculty are reported to have inadequate knowledge of HIV and hold misconceptions about caring for PLWHA [55]. Furthermore, this study proposes raising awareness of PLWHA to improve students’ willingness through contextual simulation, experience sharing, and personal contact. Cross-cultural peer interactions following HIV and AIDS didactic courses have been shown to improve students’ perceptions of PLWHA. Previous studies reported that contact-based education interventions could effectively reduce healthcare providers’ stigma towards patients with mental illness [56]. Additionally, mass-media-led preventive approaches are also recommended to increase students’ HIV awareness and perception of transmission routes [57]. As a subcategory emerging from the transcripts, nursing professionalism is beneficial in improving students’ willingness and intention to care for PLWHA. Nursing professionalism, rooted in the principles of professionalism, caring, and altruism in caring for individuals [58], is associated with nursing work intention [59], care performance, and patient safety activities [60]. These findings demonstrate the importance of enhancing HIV and AIDS education and training, establishing positive role models, and promoting nursing professionalism to adequately prepare nursing students for HIV care.

Concerningly, student nurses revealed that they did not receive hands-on HIV and AIDS training in school or specialized HIV and AIDS training in hospitals, leading them to be unprepared to interact with PLWHA confidently and effectively, which is consistent with Frain’s findings [61]. Similarly, Murwira found that HIV and AIDS content is seldom included in students’ disciplines, and few nursing faculty members are equipped to teach about HIV and AIDS in South Africa [62]. A lack of standardized training is a serious threat to providing PLWHA with effective, high-quality care [49,63]. However, student nurses have “great HIV and AIDS education and training needs”, which is supported by Murwira et al. [64], who analyzed undergraduate students’ HIV and AIDS knowledge and advocated the need for more HIV and AIDS education. Ongoing education, professional guidance, mentorship, and formative feedback are crucial for equipping caregivers with the skills to provide effective HIV and AIDS testing and counselling [65]. With the revolutionary advances in HIV treatment, student nurses are very interested in anti-HIV medication and treatment outcomes. An improved understanding of HIV and AIDS treatment will also allow student nurses to better provide quality care to PLWHA. HIV prevention strategies (e.g., safe blood supply, microbial agents and vaccines) have also attracted students’ strong interest. Given the high prevalence of psychological problems among PLWHA [66,67], psychotherapy procedures such as cognitive-behavioral therapy [68], aerobic exercise programs [69], mindfulness and acceptance-based interventions [70] could be used to address their psychosocial disorders. Our findings demonstrate the need for further psychotherapy training for student nurses. Moreover, the fear of contracting HIV through clinical practice is a common concern among healthcare providers [16,71]. In line with the findings of Suglo et al. [72], a need for HIV post-exposure reporting protocols and prophylaxis training is also illustrated by the participants. Previous studies have indicated that healthcare providers possess insufficient knowledge of pre-exposure prophylaxis and a limited understanding of the associated clinical guidelines [73]. Despite the fact that Chinese hospitals had developed appropriate post-occupational exposure management protocols, some students still stated that they were unaware of approved protocols and proper actions after needle-stick injuries, which is consistent with Al Qadire et al.’s findings [74]. It is widely agreed that injecting drug users, commercial sex workers, people with sexually transmitted diseases, and blood/plasma donors are at high risk of HIV infection [75]. Some students are curious about HIV high-risk behaviors and want to receive training on such an aspect, similar to the studies of Raia-Barjat et al. [76] and Brown, Sessanna, and Paplham [77]. This fact may emphasize the importance of preventative and control programs for HIV infection risk behaviors. More efforts are required to develop and implement comprehensive HIV and AIDS education and training programs to meet nursing students’ learning needs within nursing schools and healthcare settings.

## 6. Limitations

This study has the following limitations. First, we collected data from only 18 student nurses who had cared for patients with AIDS, which may limit the transferability of the findings, although these participants came from 14 tertiary hospitals in seven provinces. Second, small sample sizes can limit the breadth of perspectives and suggestions. Future research could recruit a larger and more diverse sample of student nurses from various sociodemographic backgrounds to enhance the generality of the results. Third, this study did not consider the perspectives of nursing educators or clinical instructors; additional efforts could be made to investigate the perspectives of nursing educators, which may provide deep insight into strategies to increase nursing students’ willingness to care for PLWHA and their educational or training needs. Fourth, qualitative methodologies have inherent limitations, such as the possibility of subjective tendency. Future quantitative investigations could enrich the findings of this study.

## 7. Conclusions

Our study reveals significant insights into improving nursing students’ willingness to care for PLWHA and identifies specific educational and training needs in HIV and AIDS care. A notable positive shift was observed in student nurses’ willingness to care for PLWHA following their AIDS-related clinical experiences, emphasizing the importance of HIV and AIDS education and training, exemplary role models, and nursing professionalism. However, this study also exposed critical training gaps regarding insufficient HIV and AIDS practice training in educational settings and a lack of specialized HIV and AIDS training in hospital environments. Critical knowledge and skills are urgently needed in HIV/AIDS treatment, prevention, PLWHA psychological support, post-exposure protocols, and risk behavior management. Our findings highlight the need for evidence-based political, health, and cultural initiatives to equip nursing students with the skills and knowledge necessary in caring for PLWHA. Recommended instructional strategies include enhancing culturally sensitive HIV and AIDS education, reducing stigma related to PLWHA, and policy priorities in HIV and AIDS education. This study provides theoretical and practical implications for educational interventions in preparing qualified nurses to meet the complex needs of PLWHA.

## Figures and Tables

**Table 1:** A formal outline to facilitate interviews with student nurses.

Topic	Open-Ended Questions
1. Willingness to care for PLWHA	(1.1) How was your willingness to provide care to PLWHA before and after your internship?
	(1.2) What specific care activities were you willing to offer to PLWHA before and after your internship?
	(1.3) What strategies do you think can be applied to improve nursing students’ willingness to deliver care to PLWHA?
2. Educational and training needs	(2.1) What HIV and AIDS education and training, including lectures, has been provided by the school or hospital?
	(2.2) What knowledge of HIV and AIDS do you expect to gain?

**Table 2:** Socio-demographic characteristics of participants.

Characteristics		Number of Participants N (%)
Gender	Female	14 (77.78)
	Male	4 (22.22)
Age (years)	21	4 (22.22)
	22	9 (50.00)
	23	5 (27.78)
Attended University	Hunan Province (Xiangnan University, Xiangxing College of Hunan University of Chinese Medicine, Hunan University of Chinese Medicine)	8 (72.22)
	Guangxi Zhuang Autonomous Region (Guangxi University of Chinese Medicine)	3 (16.67)
	Yunnan Province (Yunnan University of Business Management, Kunming Medical University Haiyuan College)	3 (16.67)
	Tibet Autonomous Region (Tibet University)	2 (11.11)
	Sichuan Province (North Sichuan Medical College)	1 (5.56)
	Guizhou Province (Guizhou Medical University)	1 (5.56)
Internship Hospital	Hunan Province (Xiangya Second Hospital of Central South University, Chenzhou First People’s Hospital, The First Affiliated Hospital of Hunan University of Chinese Medicine Zhuzhou Central Hospital)	5 (27.78)
	Guangxi Zhuang Autonomous Region (Nanning Second People’s Hospital, People’s Hospital of Guangxi Zhuang Autonomous Region, The First Affiliated Hospital of Guangxi University of Chinese Medicine)	3 (16.67)
	Yunnan Province (The First Affiliated Hospital of Kunming Medical University, The Third People’s Hospital of Yunnan Province)	3 (16.67)
	Tibet Autonomous Region (People’s Hospital of Tibet Autonomous Region)	2 (11.11)
	Guangdong Province (Guangdong Zhongshan Bo’ai Hospital, Zhuhai Hospital of Guangdong Provincial Hospital of Traditional Chinese Medicine)	3 (16.67)
	Sichuan Province (Affiliated Hospital of North Sichuan Medical College)	1 (5.56)
	Guizhou Province (Affiliated Hospital of Guizhou Medical University)	1 (5.56)
The number of AIDS patients	1	4 (22.22)
	2	10 (55.56)
	3	2 (11.11)
	4	1 (5.56)
	8	1 (5.56)
Frequency of participation in AIDS-related training (times)	0	3 (16.67)
	1	7 (38.89)
	2	6 (33.33)
	3	2 (11.11)

**Table 3:** Superordinate themes, subordinate themes, and subcategories in the interviews.

Super-Ordinate Themes	Subordinate Themes	Subcategories	No. of Responses (N = 18)
Student nurses’ willingness to care for PLWHA	Expressions of care willingness	Unexpecting to care for PLWHA before internship	10
		Strong willingness to care for PLWHA in future work	18
	Methods of improving care willingness	Increase HIV and AIDS education and training	13
		Make psychological preparations	10
		Set positive role models	7
		Raise awareness of PLWHA	6
		Foster nursing professionalism	4
HIV and AIDS-related Educational and training programs	Inadequate HIV and AIDS education and training frequency	Lack of hands-on HIV and AIDS training in school	7
		Lack of specialized HIV and AIDS training in hospital	14
	Salient HIV and AIDS education and training needs	Progress in HIV and AIDS treatment	8
		HIV-prevention strategies	7
		Psychological support skills for PLWHA	7
		HIV post-exposure reporting protocols and prophylaxis	6
		HIV-infection risk behaviors	3

## Data Availability

The data presented in this study are available on request from the corresponding author.

## Abbreviations

HIV: human immunodeficiency virus; AIDS: acquired immunodeficiency syndrome; PLWHA: people living with HIV and AIDS
